# Clinician-Led Code-Free Deep Learning for Detecting Papilledema and Pseudopapilledema Using Optic Disc Imaging

**DOI:** 10.1167/tvst.15.2.25

**Published:** 2026-02-20

**Authors:** Riddhi Shenoy, Gurtek Singh Samra, Rishi Sekhri, Ha-Jun Yoon, Seema Teli, Ian DeSilva, Zhanhan Tu, Gail DE Maconachie, Mervyn G Thomas

**Affiliations:** 1 Ulverscroft Eye Unit, School of Psychology and Vision Sciences, College of Life Sciences, University of Leicester, Leicester, UK; 2 Department of Ophthalmology, University Hospitals of Leicester, Leicester Royal Infirmary, Leicester, UK; 3 School of Allied Health Professionals, Nursing and Midwifery, Faculty of Health, University of Sheffield, Sheffield, UK

**Keywords:** AutoML, papilloedema, optic disc drusen

## Abstract

**Purpose:**

Differentiating pseudopapilledema from papilledema is challenging, but critical for prompt diagnosis and to avoid unnecessary invasive procedures. This study evaluates automated machine learning (AutoML) model performance for distinguishing the presence and severity of papilledema using near infrared reflectance images obtained from standard optical coherence tomography, comparing the performance of different AutoML platforms.

**Methods:**

A retrospective cohort study was conducted using University Hospitals of Leicester, NHS Trust data. Optic nerve head-centered OCT imaging was obtained for 289 patients (813 images) from 2021 to 2024, with normal optic discs (69 patients, 185 images), papilledema (135 patients, 372 images), and optic disc drusen (ODD) (85 patients, 256 images). AutoML platforms—Amazon Rekognition, Medic Mind, and Google Vertex—were evaluated for (1) distinguishing papilledema from normal discs and ODD and (2) grading papilledema severity (mild/moderate vs. severe). Model performance was evaluated using area under the curve (AUC), precision, recall, F1 score, and confusion matrices for all six models.

**Results:**

Amazon Rekognition showed the best performance in distinguishing papilledema from normal/ODD (AUC, 0.90; F1 score, 0.81) and grading severity of papilledema (AUC, 0.90; F1 score, 0.79), outperforming Google Vertex and Medic Mind, which had slightly lower accuracy and higher misclassification rates.

**Conclusions:**

This evaluation demonstrates the feasibility of AutoML platforms in papilledema classification using near-infrared reflectance images obtained from standard optical coherence tomography. Further external validation is needed to confirm clinical utility.

**Translational Relevance:**

Automated machine learning can be feasibly used to provide an accessible, scalable solution for clinical teams without coding expertise to recognize and characterize papilledema.

## Introduction

Papilledema, the swelling of the optic disc owing to raised intracranial pressure, is a critical finding in the diagnosis of various neurological conditions, including idiopathic intracranial hypertension, brain tumors, and venous sinus thrombosis. Early detection is essential to prevent associated morbidity or mortality and to guide appropriate medical intervention.^1^^,^^2^ However, distinguishing papilledema from pseudopapilledema, which can be caused by conditions such as optic disc drusen (ODD), is clinically challenging.^3^ Accurate diagnosis is crucial to avoid unnecessary invasive procedures, such as lumbar punctures or neuroimaging, which carry risks and financial costs, and to ensure appropriate management for patients.

After establishing a diagnosis of papilledema, its severity is documented using the Frisén grading system, which is used alongside patient symptomatology and neuroimaging to dictate the urgency of intervention and subsequent therapeutic response. Frisén grading is performed using retinal photographs or on ophthalmoscopy and has five grades based on severity of disc swelling.^4^ The clinical utility of accurate Frisén grading has been well-documented, with higher grades at presentation associated with worse visual function at baseline and follow-up.^5^ However, its reproducibility is limited, with inter-rater agreement as low as 49%, leading to significant variability in clinical practice.^4^ Clinically, a retinal photograph may not be obtained for most patients presenting to ophthalmology, whereas optical coherence tomography (OCT) is now ubiquitous in ophthalmological assessment and also commonly obtained at high street opticians. Artificial intelligence has been posed as a solution for consistent and scalable grading, with validation studies of bespoke deep learning models to identify papilledema showing performance comparable with human experts.^6^^–^^9^

Currently, most commercial OCT devices incorporate a high-contrast near-infrared reflectance (NIR) image acquired in combination with the structural OCT.^10^ NIR images are noninvasive and provide an en face composite image of multiple B-scan OCTs, which can be viewed as a fundus image and be used to grade papilledema using the Frisén system.^10^ NIR images can also be easily obtained through an undilated pupil and can deliver adequate image quality, even in poor signal quality and significant disc oedema.^10^^–^^12^

The advent of automated machine learning (AutoML), also known as code-free artificial intelligence, offers a promising solution to overcome the limitations of traditional grading systems. AutoML platforms enable the development of machine learning models for image classification without requiring expertise in coding, making them an attractive option for clinicians who are not versed in machine learning methods.^13^ Different AutoML platforms for classifying retinal photographs and OCT images have been shown to have varying performance and different features that increase usability, such as customizing testing and training data splits.^14^

This study is the first to demonstrate the feasibility of AutoML to develop a model to distinguish pseudopapilledema from papilledema using NIR images and compare the performance across different AutoML providers.

## Methods

### Dataset and Image Acquisition

Optic nerve head–centered OCT and NIR images were acquired using the Heidelberg SPECTRALIS OCT imaging system (Heidelberg Engineering, Heidelberg, Germany) at the University Hospitals of Leicester NHS Trust between 2021 and 2024. Images were obtained under standardized acquisition protocols (acquisition software version 6.16.8.0), primarily employing two scanning approaches. The first method used was peripapillary ring scans with a circle diameter of 12° consisting of 768 A-scans per circle. The automatic real-time tracking mode was enabled, averaging 100 images to enhance image quality. These scans achieved axial scaling (*z* axis) of 3.87 µm/pixel and transverse scaling (*x* axis) of approximately 15.13 µm/pixel. The second method used was peripapillary volume scans acquired under the high-speed mode, typically with a 15° × 15° volume pattern, comprising 37 horizontal B-scans. Automatic real-time tracking averaging of 54 images per B-scan was enabled. Enhanced depth imaging was only used in suspected buried drusen cases. NIR images were obtained simultaneously with OCT scans with the following parameters: scan angle of 30°, image dimensions of 768 × 768 pixels (covering ∼8.1–10.9 mm², depending on axial length), transverse scaling of 10.61 to 14.15 µm/pixel, automatic real-time tracking averaging of 45 frames per image, IR laser power at 100%, sensitivity (DC/DC) set between 81% and 90%, and auto-brightness enabled. All images were initially stored in the .E2E format, the proprietary file format used by the Heidelberg Engineering. To facilitate further analysis, these files were converted to PNG fundus images using the OCT-Convert Python package.

Patients were retrospectively identified using *International Classification of Diseases*, 10th edition, codes and electronic medical records. Diagnoses of papilledema and ODD were confirmed clinically and verified through expert review of OCT B-scans and comprehensive medical record review. Longitudinal OCT and NIR images were included to represent varying Frisén grades, ensuring no duplication of identical grades for the same patient. Controls consisted of patients referred for ophthalmic evaluation unrelated to optic nerve pathology (e.g., posterior vitreous detachment, floaters). Detailed medical record review and OCT analysis confirmed no evidence of optic nerve pathology. For simplicity, this group is referred to as the normal cohort throughout the study. The ground-truth dataset consisted of data from a total of 289 patients (813 images). Sixty-nine patients (185 images) were labelled normal, 85 patients (256 images) were labelled ODD, and 135 patients (372 images) were labelled papilledema. This study uses data from an institutional database previously analysed for OCT biomarkers by Sekhri et al. (2025),^15^ although with an extended timeframe (2021–2024 vs. 2021–2022) and expanded cohort (289 vs. 213 patients). In that analysis, peripapillary hyper-reflective ovoid mass-like structures were present in 65% of papilledema cases and 68% of ODD cases, consistent with the literature showing peripapillary hyper-reflective ovoid mass-like structures as a nonspecific marker of optic nerve head pathology rather than a discriminating feature between these conditions.^15^ (See Supplementary Table S1 for demographic information.)

### Papilledema Grading

Images with papilledema were graded using the Frisén system by three independent graders. A consensus grade was reached for each image, and severity was classified as mild/moderate (Frisén grades 1–3) or severe (Frisén grades 4–5). This grouped severity classification has been described in similar studies.^9^ The severity labels were used to train the AutoML models.

### Training and Testing

**Figure 1.**
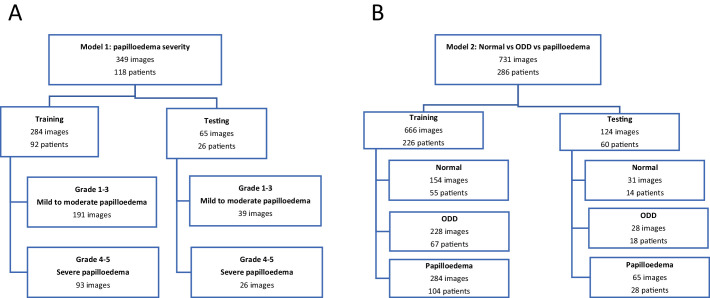
(**A**) training and testing dataset split for model 1: papilledema severity mild/moderate vs. severe and (**B**) training and testing dataset split for model 2, differentiating papilledema vs. normal disc vs. ODD.

Patients were randomly split into training (80%) and testing (20%) sets, ensuring that images from the same patient did not exist in different sets to develop two models. Testing and training data splits are shown in Figure 1, where model 1 aimed to differentiate severity of papilledema using the modified grading (Fig. 1A) and model 2 aimed to differentiate papilledema from ODD and normal optic discs (Fig. 1B).

### Code-Free Machine Learning Platform

The clinical research team with limited previous machine learning coding experience performed the AutoML model development. Amazon Rekognition (AWS), Medic Mind, and Google Vertex platforms were selected for their ability to manually customize the training and testing data splits, which allowed data to be split at the patient level and prevented images from the same patient being used for testing and training the model. Furthermore, these platforms have previously been shown to perform well in image classification tasks using fundus photographs and OCT images.^14^ AWS and Google Vertex platforms use proprietary neural architecture search to automatically select optimal algorithms. MedicMind offers a choice of algorithms, from which EfficientNet was selected for previously documented strong accuracy–efficiency performance.^16^ All platforms used transfer learning, leveraging models pretrained on large natural image datasets and fine tuning on our ophthalmic data. The models were evaluated using a custom script. These platforms only provide a confidence score for each class and the precision, recall, F1 score, and area under the curve (AUC) are derived from this score.

### Ethics Approval

All data in this study were retrospectively collected, with necessary ethical approval (IRAS Project ID: 261121; REC Reference: 20/EM/0040).

## Results

### Model 1: Papilledema Severity Grading

**Figure 2.**
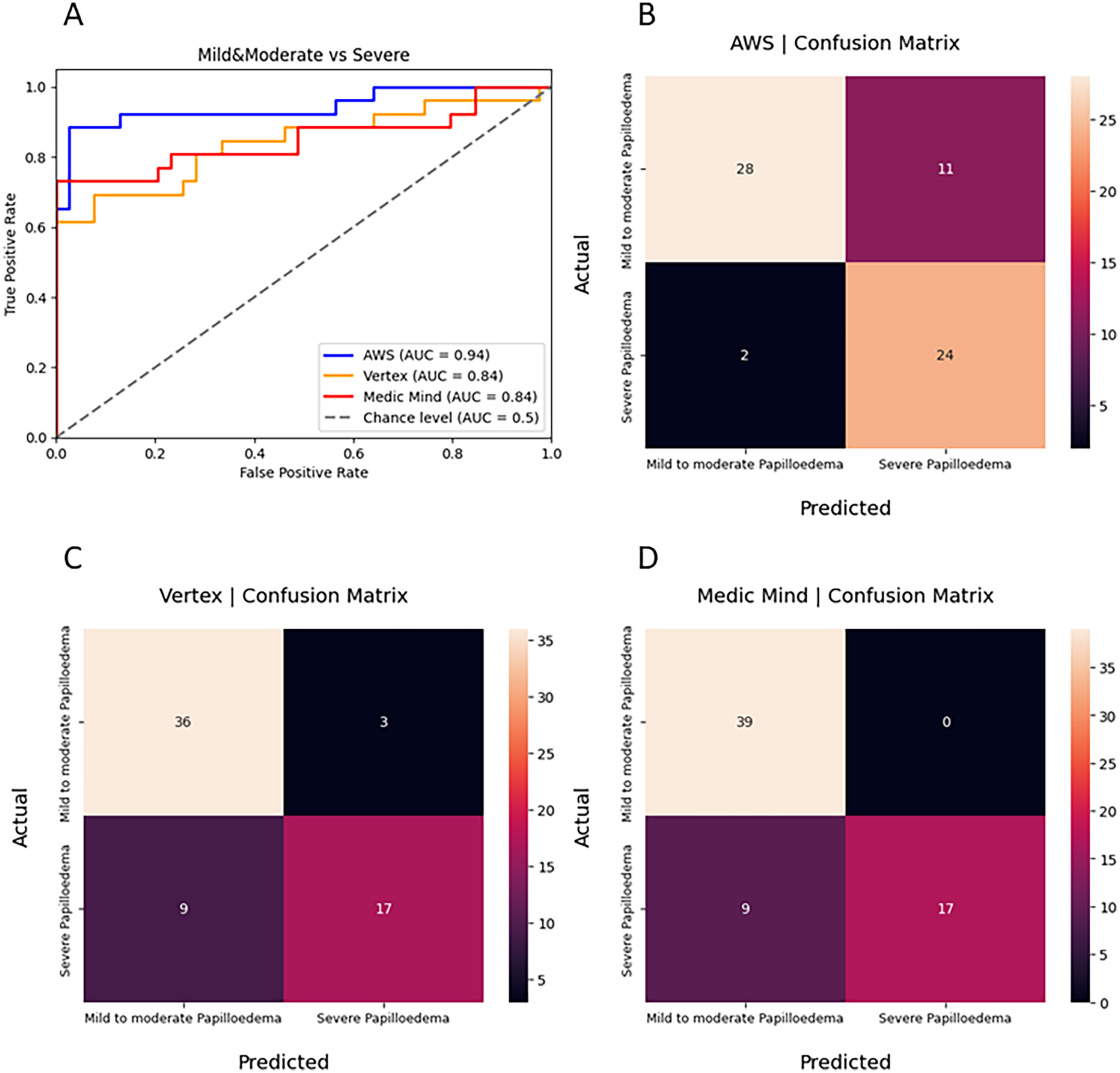
(**A**) AUC curves of model 1 performance between Medic Mind, Google Vertex, and AWS AutoML models in classifying papilledema severity (mild/moderate vs. severe), confusion matrices for model 1 on (**B**) AWS, (**C**) Google Vertex, and (**D**) Medic Mind.

All three models performed well in differentiating between mild to moderate and severe papilledema. The AWS model performed the best (AUC, 0.94; F1 score, 0.78), followed by Google Vertex (AUC, 0.84; F1 score, 0.74) and Medic Mind (AUC, 0.82; F1 score, 0.79) (Table). AWS most frequently misclassified mild to moderate papilledema as severe papilledema; however, Google Vertex and Medic Mind most frequently misclassified severe papilledema as mild to moderate papilledema (Fig. 2).

**Table.** Performance Metrics Including F1 Score, Precision, and Recall for Model 1 and Model 2

	F1	Precision	Recall
Model 1: Mild/moderate vs. severe papilledema			
AWS	0.79	0.69	0.92
Google Vertex	0.74	0.85	0.65
Medic Mind	0.79	1.0	0.65
Model 2 papilledema vs. ODD vs. normal optic disc			
AWS	0.81	0.81	0.84
Google Vertex	0.73	0.74	0.74
Medic Mind	0.72	0.74	0.78

### Model 2: Papilledema vs. ODD vs. Normal

**Figure 3.**
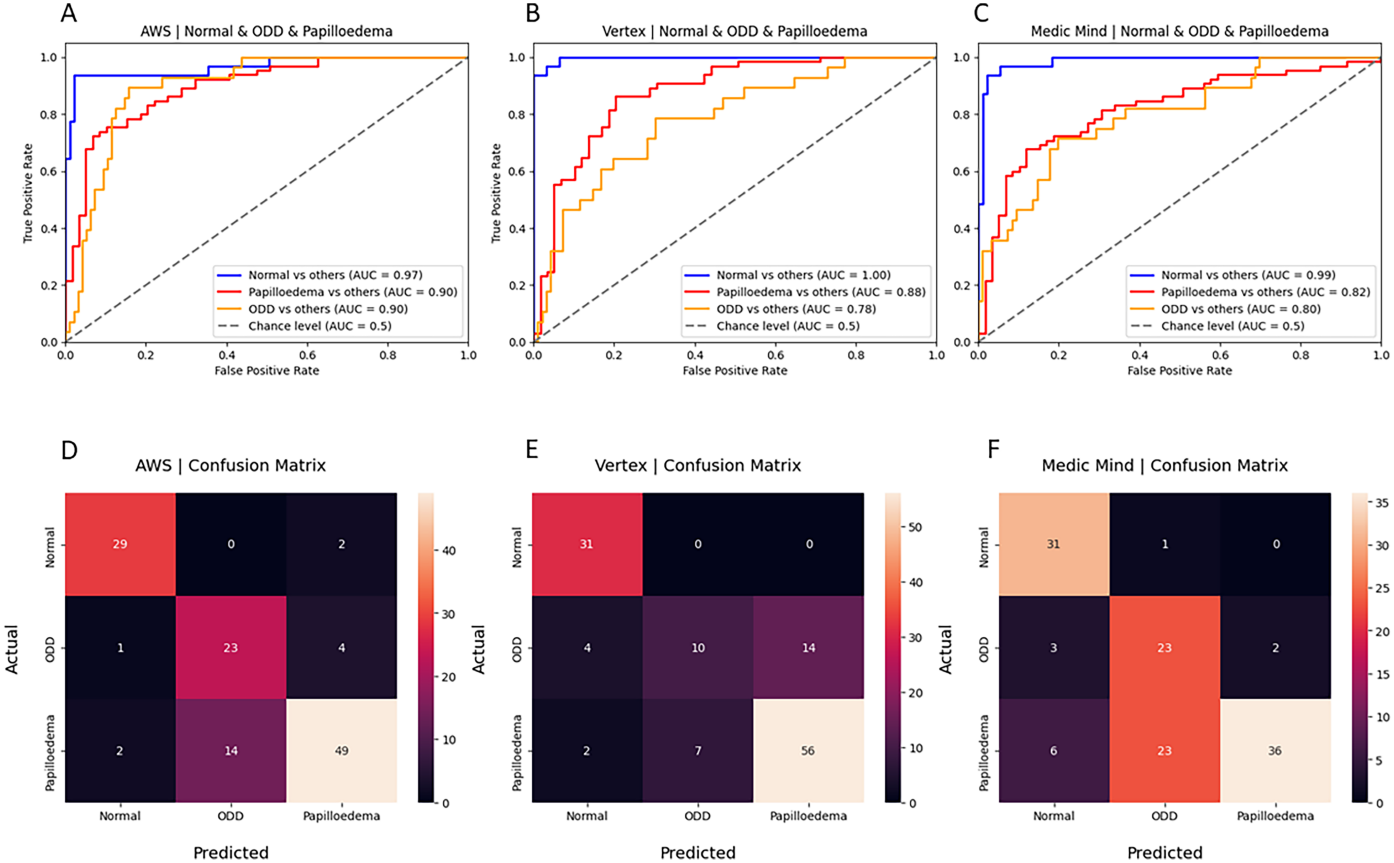
AUC curves of performance in classification model 2 (papilledema vs. normal optic discs vs. ODD) for each platform (**A**) AWS, (**B**) Google Vertex, and (**C**) Medic Mind with corresponding confusion matrices for each model, (**D**) AWS, (**E**) Google Vertex, and (**F**) Medic Mind.

All three models demonstrated good performance in differentiating between normal optic discs, ODD, and papilledema in the multiclass model 2. AWS showed the best performance overall (average AUC, 0.92; F1 score, 0.81), closely followed by Google Vertex (average AUC, 0.87; F1 score, 0.73) and Medic Mind (average AUC, 0.87; F1 score, 0.72) (Table). Overall, the models from all three platforms showed the best performance in identifying normal optic discs (Fig. 3). Considering accurate identification of papilledema would likely have the greatest clinical utility, AWS also showed the best performance in differentiating papilledema (AUC, 0.90), compared with Google Vertex (AUC, 0.88) and Medic Mind (AUC, 0.82) (Fig. 3).

**Figure 4.**
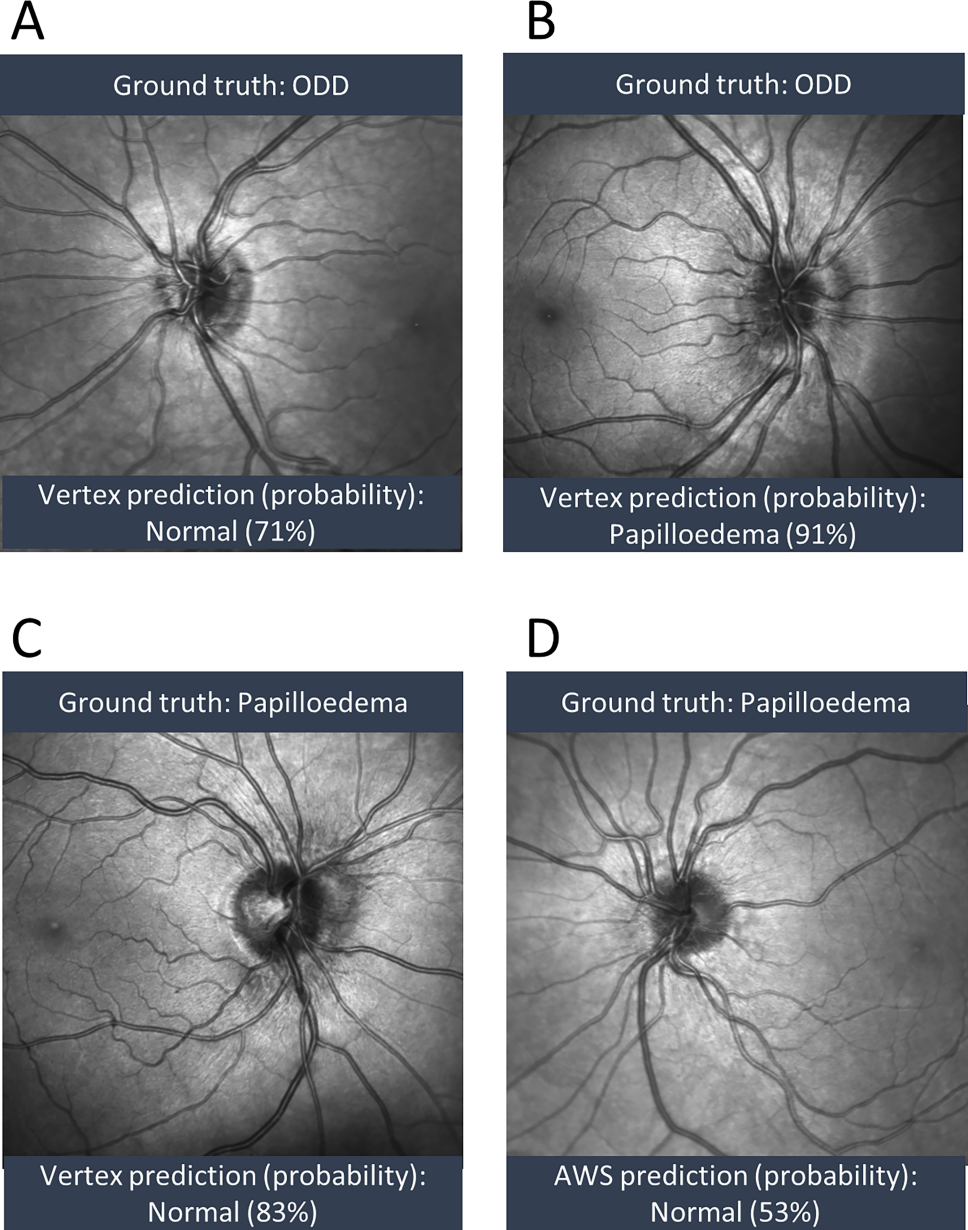
(**A–D**)examples of misclassified images with ground truth label and predicted label and model probability of prediction.

The models most frequently misclassified ODD as papilledema or normal optic discs (Figs. 4A, 4B). Whereas both AWS and Google Vertex misclassified 2 of 65 papilledema images as normal optic discs, Medic Mind misclassified 6 papilledema images as normal (Fig. 3). Images of papilledema misclassified as normal showed mild or moderate papilledema (Fig. 4C), with one showing mild papilledema in a patient who previously had severe papilledema (Fig. 4D).

## Discussion

This study is the first to demonstrate the feasibility of using AutoML platforms to classify and grade papilledema from NIR OCT images. In this study, two models have successfully been developed using three different AutoML platforms—AWS, Google Vertex, and Medic Mind—to improve recognition and characterization of papilledema using NIR images. Across all three platforms, the binary classification model was reliably able to predict severity of papilledema and the multiclass model showed good reliability in differentiating papilledema from normal optic discs, but performed least reliably in predicting ODD from papilledema with higher rates of misclassification.

To our knowledge, there is no published comparison of AutoML models for classifying papilledema based on NIR images. Although previous studies have shown that custom-engineered deep learning systems (DLSs) can achieve high accuracy (AUC, 0.89–0.99) in detecting papilledema from fundus images, developing a DLS requires significant computational resources, technical expertise, and extensive annotated datasets, which limit their widespread implementation.^7^^,^^8^^,^^17^^–^^19^

Difficulty in classifying papilledema using Frisén grading has been noted, particularly surrounding the lack of reproducibility and utility in monitoring optic disc changes.^20^^,^^21^ Indeed, in clinical practice, the simplified classification of papilledema used in this study may often be used in clinical decision-making.^9^ Fundus findings on examination may also be triangulated with patient symptoms and other imaging modalities, including quantitative and qualitative findings on transverse axial OCT images, and ultrasonography findings.^22^

Although AutoML models have been shown to be comparable with bespoke DLSs in ophthalmology, it is unclear how the inherent challenges of using the Frisén grading scale are reflected in the AutoML model performance, which is reliant on labelled data by human graders.^23^ When training the BONSAI model to determine severity of papilledema, moderate papilledema (Frisén grade 3) was most frequently misclassified as severe papilledema, and misclassified cases of severe papilledema contained other optic disc pathologies such as cotton wool spots or hemorrhages.^9^ In our study, the AutoML models performed well with high AUCs for classifying mild to moderate vs. severe papilledema, although there was variability in misclassifications between platforms.

Image type and quality may impact model performance, although the BONSAI DLS has been shown to have comparable performance in both dilated fundus photographs and undilated fundus photographs, obtained by emergency department professionals, with 84% sensitivity and 99% specificity for differentiating papilledema from normal optic discs and ODD.^17^^,^^18^ To ensure sufficient image quality, further DLS models have been proposed to classify image quality of optic discs in fundus photographs.^24^

As a part of data curation for model development, images with decentration or poor quality are excluded as ungradable. The original validation study of the BONSAI DLS excluded 153 photographs deemed ungradable, with 15,846 images subsequently included.^17^ In comparison, a much higher rate of ungradable images was reported in the study using undilated fundus photographs obtained by nurses or students in the emergency department, where 463 patients’ images were excluded from the original 1291 patients owing to poor quality image or missing the optic disc.^18^

Conversely, the low rate of ungradable images in our study is notable, attesting to the reliable quality of NIR image generated by commercial OCT scans. These findings could support the use of NIR images to assess the optic disc in low-resource settings, where one machine could reliably produce images of sufficient quality for both transverse axial and en face images of the retina. Moreover, the use of NIR images provides a compelling case for their utility in both high-resource and low-resource settings. These images, with no flash or pupil dilation requirements, are particularly advantageous for pediatric patients or individuals with photophobia, where traditional fundus photography may fail owing to artefacts like blink shadows or iris silhouettes.

The strengths of this study include manual data splitting at the patient level, using longitudinal data, and having a multirater consensus to establish ground truth labels. Ensuring images from the same patient did not appear in both training and testing sets reduced the risk of overestimation of the model performance and prevented inadvertent data leakage between sets. Using longitudinal data, ensuring only different Frisén-graded images were used for any single patient, enhanced the sample size without duplicating images of the same grade, which could otherwise lead to model overfitting.

Limitations of the dataset include a relatively small sample size, class imbalance, and lack of external validation. The sample may not have captured the breadth of phenotypes of optic discs, ODD and papilledema. Furthermore, the ODD class had the smallest sample size, which could have contributed to the model's performance for this class. The size of the dataset may have also contributed to model overfitting, which would need to be evaluated by testing on a new, unseen dataset. AutoML platforms are known to have poor explainability, and the selected platforms did not offer an integrated function for generating saliency maps.^14^ Future developments in machine learning may enable innovative tools to facilitate the interpretation of AutoML. External validation was not carried out on this model, which would be necessary to assess the utility of the models in different clinical settings.

Although the literature suggests OCT RNFL thickness is valuable in monitoring the longitudinal progression of papilledema in people with idiopathic intracranial hypertension, there are currently no clinical guidelines in the UK for either monitoring or assessing severity of papilledema using RNFL thickness.^25^ Future work in this area could apply machine learning to explore the utility and feasibility of different OCT quantitative biomarkers in the longitudinal monitoring of papilledema in a diagnosed patient.

This study highlights the feasibility and potential of AutoML platforms in grading papilledema severity and classifying NIR images into normal, ODD, and papilledema categories. By providing accessible tools for artificial intelligence model development, AutoML has the potential to transform the diagnostic landscape of neuro-ophthalmology, particularly in settings with limited resources, combined with clinician input to detect misclassified true papilledema. Future research should focus on addressing dataset limitations and externally validating the models to assess generalizability and maximize clinical impact.

## Supplementary Material

Supplement 1

## References

[1] Malmqvist L, Bursztyn L, Costello F, et al. The Optic Disc Drusen Studies Consortium recommendations for diagnosis of optic disc drusen using optical coherence tomography. *J Neuroophthalmol*. 2018; 38(3): 299–307. 29095768 10.1097/WNO.0000000000000585

[2] Merchant KY, Su D, Park SC, et al. Enhanced depth imaging optical coherence tomography of optic nerve head drusen. *Ophthalmology*. 2013; 120(7): 1409–1414. 23531353 10.1016/j.ophtha.2012.12.035

[3] Mollan SP, Markey KA, Benzimra JD, et al. A practical approach to, diagnosis, assessment and management of idiopathic intracranial hypertension. *Pract Neurol*. 2014; 14(6): 380–390. 24809339 10.1136/practneurol-2014-000821 PMC4251443

[4] Frisén L . Swelling of the optic nerve head: a staging scheme. *J Neurol Neurosurg Psychiatry*. 1982; 45(1): 13–18. 7062066 10.1136/jnnp.45.1.13 PMC491259

[5] Micieli JA, Bruce BB, Vasseneix C, et al. Optic nerve appearance as a predictor of visual outcome in patients with idiopathic intracranial hypertension. *Br J Ophthalmol*. 2019; 103(10): 1429–1435. 30530819 10.1136/bjophthalmol-2018-313329

[6] Jiang Z, Biousse V, Wong TY, et al. Artificial intelligence to detect papilledema from ocular fundus photographs. *N Engl J Med*. 2020; 382(18): 1687–1695. 32286748 10.1056/NEJMoa1917130

[7] Biousse V, Newman NJ, Najjar RP, et al. Optic disc classification by deep learning versus expert neuro-ophthalmologists. *Ann Neurol*. 2020; 88(4): 785–795. 32621348 10.1002/ana.25839

[8] Lin MY, Najjar RP, Tang Z, et al. The BONSAI (Brain and Optic Nerve Study with Artificial Intelligence) deep learning system can accurately identify pediatric papilledema on standard ocular fundus photographs. *J AAPOS*. 2024; 28(1): 103803. 38216117 10.1016/j.jaapos.2023.10.005

[9] Vasseneix C, Najjar RP, Xu X, et al. Accuracy of a deep learning system for classification of papilledema severity on ocular fundus photographs. *Neurology*. 2021; 97(4): e369–e377. 34011570 10.1212/WNL.0000000000012226 PMC8362357

[10] Vaz-Pereira S, Monteiro-Grillo M, Engelbert M. Near-infrared reflectance imaging of neovascularization in proliferative diabetic retinopathy. *Int J Retina Vitreous*. 2020; 6(1): 59. 33292751 10.1186/s40942-020-00263-8 PMC7682524

[11] Carey AR, Bosley TM, Miller NR, McCulley TJ, Henderson AD. Use of en face optical coherence tomography to monitor papilledema in idiopathic intracranial hypertension: a pilot study. *J Neuroophthalmol*. 2021; 41(2): 212–216. 32235232 10.1097/WNO.0000000000000940

[12] Aojula A, Mollan SP, Horsburgh J, et al. Segmentation error in spectral domain optical coherence tomography measures of the retinal nerve fibre layer thickness in idiopathic intracranial hypertension. *BMC Ophthalmol*. 2018; 17(1): 257. 29298687 10.1186/s12886-017-0652-7 PMC6389234

[13] Faes L, Wagner SK, Fu DJ, et al. Automated deep learning design for medical image classification by health-care professionals with no coding experience: a feasibility study. *Lancet Digital Health*. 2019; 1(5): e232–e242. 33323271 10.1016/S2589-7500(19)30108-6

[14] Korot E, Guan Z, Ferraz D, et al. Code-free deep learning for multi-modality medical image classification. *Nature Machine Intelligence*. 2021; 3(4): 288–298.

[15] Sekhri R, Kuht HJ, Tu Z, et al. Identifying biomarkers for papilledema and pseudopapilledema. *Sci Rep*. 2025; 15(1): 24847. 40640273 10.1038/s41598-025-09778-2 PMC12246050

[16] Tan M, Le Q. Efficientnet: Rethinking model scaling for convolutional neural networks. *International Conference on Machine Learning*; PMLR; Long Beach, California, June 9–15, 2019.

[17] Dan M, Najjar Raymond P, Zhubo J, et al. Artificial intelligence to detect papilledema from ocular fundus photographs. *N Engl J Med*. 2020; 382(18): 1687–1695. 32286748 10.1056/NEJMoa1917130

[18] Biousse V, Najjar RP, Tang Z, et al. Application of a deep learning system to detect papilledema on nonmydriatic ocular fundus photographs in an emergency department. *Am J Ophthalmol*. 2024; 261: 199–207. 37926337 10.1016/j.ajo.2023.10.025

[19] Chang MY, Heidary G, Beres S, et al. Artificial intelligence to differentiate pediatric pseudopapilledema and true papilledema on fundus photographs. *Ophthalmol Sci*. 2024; 4(4): 100496. 38682028 10.1016/j.xops.2024.100496 PMC11046195

[20] Sinclair AJ, Burdon MA, Nightingale PG, et al. Rating papilloedema: an evaluation of the Frisén classification in idiopathic intracranial hypertension. *J Neurol*. 2012; 259(7): 1406–1412. 22237821 10.1007/s00415-011-6365-6

[21] Fischer WS, Wall M, McDermott MP, Kupersmith MJ, Feldon SE, NORDIC Idiopathic Intracranial Hypertension Study Group. Photographic Reading Center of the Idiopathic Intracranial Hypertension Treatment Trial (IIHTT): methods and baseline results. *Invest Ophthalmol Vis Sci*. 2015; 56(5): 3292–3303. 26024112 10.1167/iovs.15-16465 PMC4453296

[22] Sibony PA, Kupersmith MJ, Kardon RH. Optical coherence tomography neuro-toolbox for the diagnosis and management of papilledema, optic disc edema, and pseudopapilledema. *J Neuroophthalmol*. 2021; 41(1): 77–92. 32909979 10.1097/WNO.0000000000001078 PMC7882012

[23] Wong CYT, O'Byrne C, Taribagil P, Liu T, Antaki F, Keane PA. Comparing code-free and bespoke deep learning approaches in ophthalmology. *Graefe's Arch Clin Exp Ophthalmol*. 2024; 262(9): 2785–2798. 38446200 10.1007/s00417-024-06432-x PMC11377500

[24] Bouris E, Davis T, Morales E, Grassi L, Salazar Vega D, Caprioli J. A neural network for automated image quality assessment of optic disc photographs. *J Clin Med*. 2023; 12(3): 1217. doi:10.3390/jcm12031217. 36769865 PMC9917571

[25] Vijay V, Mollan SP, Mitchell JL, et al. Using optical coherence tomography as a surrogate of measurements of intracranial pressure in idiopathic intracranial hypertension. *JAMA Ophthalmol*. 2020; 138(12): 1264–1271. 33090189 10.1001/jamaophthalmol.2020.4242 PMC7582233

