# Supplementary material for: Clinician-Led Code-Free Deep Learning for Detecting Papilledema and Pseudopapilledema Using Optic Disc Imaging
Source: Transl Vis Sci Technol. 2026 Feb 20;15(2):25. doi: 10.1167/tvst.15.2.25 (PMC12927425; doi:10.1167/tvst.15.2.25)
Supplement: Supplement 1 [file tvst-15-2-25_s001.pdf]

Supplementary Material

Supplementary table S1: demographic information for patients included in the ground-truth dataset

	Papilloedema	*ODD*	*Controls*
**Total Participants**	135	85	69
**Age (Mean ± SD)**	30.2 ± 12.4	25.3 ± 14.7	49.4 ± 19.0
**M:F Ratio**	23:112	33:52	31:38
**Ethnicity Distribution**	White: 106, Asian: 18, Mixed: 6, Other: 3, Black: 2	White: 78, Asian: 6, Mixed: 1	White: 35, Asian: 16, Black: 14, Mixed: 3, Other: 1
